# An Invisible Skin Marker for External Beam Radiation Therapy: Utilization of Ultraviolet Fluorescent Marker Pens

**DOI:** 10.7759/cureus.34347

**Published:** 2023-01-29

**Authors:** Atsuto Katano, Yuki Nozawa, Toshikazu Imae, Hideomi Yamashita, Keiichi Nakagawa

**Affiliations:** 1 Radiology, The University of Tokyo Hospital, Tokyo, JPN; 2 Department of Comprehensive Radiation Oncology, The University of Tokyo, Tokyo, JPN

**Keywords:** radiation oncology, fluorescent, ultraviolet light, dye marking, stress experiences

## Abstract

Radiation therapy plays an important role in cancer treatment along with surgery and systemic therapy. The total dose of radiation therapy is divided into small doses, and the treatment is typically delivered once a day. The total treatment period can need several weeks or more, and it is necessary to deliver the radiation dose to the target volume within the patient precisely each time. Therefore, the reproducibility of patient positioning is essential for the precision of the dose delivery. Although radiological technologies such as image-guided radiation therapy have also recently been widely used for positioning patients, skin marking is still widely used in many facilities. Skin marking is an inexpensive and universal positioning technique in patients undergoing radiation therapy; however, it is considered a major source of psychological stress. We propose the use of fluorescent ink pens, which are invisible in standard room lighting, as skin markers for radiotherapy. The primary technique of fluorescence emission is widely employed in molecular biological experiments and for assessing cleaning protocols for infection control. This technique may reduce the stress induced by skin markings during radiotherapy.

## Introduction

With the growing incidence of cancer worldwide, an estimated 10.0 million cancer-related deaths were recorded in 2020, according to the latest survey of the International Agency for Research on Cancer [1]. Radiation therapy plays an important role in cancer treatment, with 50% of patients undergoing radiation therapy during the course of treatment [2]. The reproducibility and accuracy of human body positioning can critically impact radiation therapy. Although image-guided radiation therapies (IGRTs) and surface-guided radiation therapies (SGRTs) have been recently explored [3], skin marking remains a frequent complementary method.

However, skin markings can induce unpleasant emotional reactions in patients undergoing radiotherapy. Asada et al. conducted questionnaire-based surveys among patients with cancer who underwent radiotherapy [4]. The authors reported that of 105 patients who were undergoing radiotherapy and completed the survey, 59 (56.2%) had uncomfortable emotional experiences. Among the 59 patients, 26 (44.1%) endured dirty clothes owing to the application of skin markers, and 13 (22.0%) sustained widespread marking areas or colored markings.

## Technical report

In general, a planning computed tomography (CT) scan is done to acquire three-dimensional images for treatment planning before starting external beam radiotherapy. Once the patient's set-up is determined at the planning CT scan, skin markers are drawn on the patient’s body using oil-based markers to identify the location of the patient. This skin marking must remain until the entire radiation therapy is completed to accurately reproduce the patient’s position, which causes cosmetic and psychological problems.

**Figure 1:**
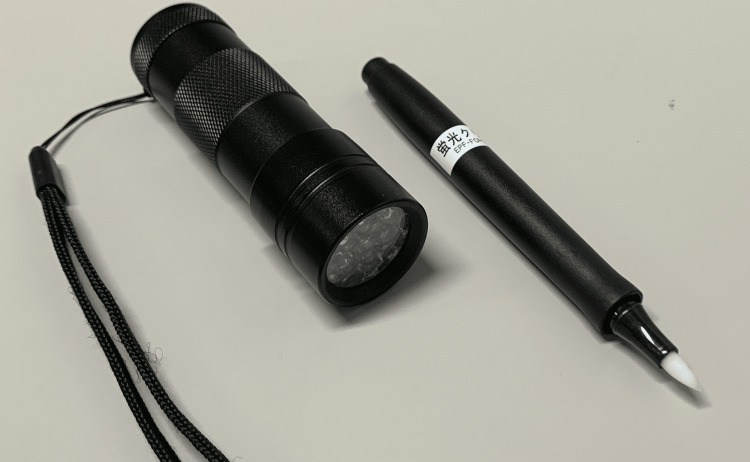
Skin marker system. Ultraviolet light-emitting diode torch (left) and fluorescent ink marker (right) provided by Shachihata Inc. (Nagoya, Aichi, Japan).

Our proposal is the use of fluorescent ink pens as an invisible skin marker during radiotherapy. The principal technologies of fluorescence emission are widely utilized in molecular biology experiments, as well as to confirm cleaning protocols in infection control [5]. Our skin marker system comprises the following two components: a fluorescent ink marker and an ultraviolet (UV) light-emitting diode (LED) torch (Figure 1), both provided by Shachihata Inc. (Nagoya, Aichi, Japan).

**Figure 2:**
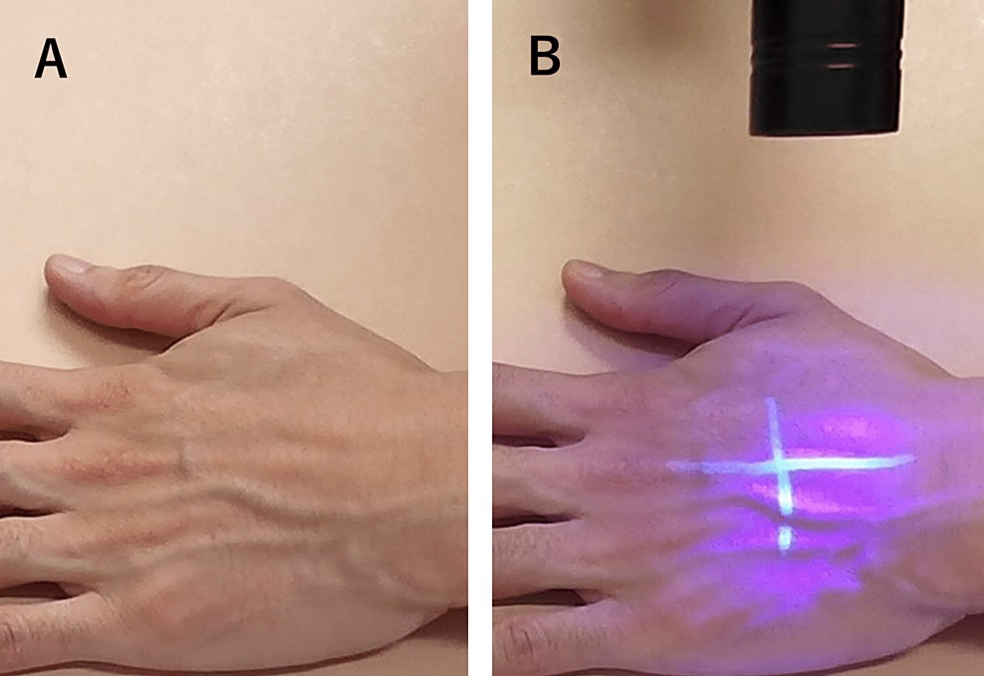
A line drawn using the fluorescent ink marker. The line is invisible under standard room lighting (A). The depicted area was irradiated using a light-emitting diode torch (upper side). The line is clearly visible to the human eye (B).

The line drawn using the fluorescent ink marker was invisible under standard room lighting (Figure 2A). To confirm the presence of the line, the depicted area was irradiated with LED light, and the line could be visualized by the human eye (Figure 2B). The photoemission mechanism underlying this phenomenon can be explained by the transition of an electron between two different energy levels.

The component of the fluorescent ink marker possesses a thiophene-based structure, which emits fluorescence upon excitation by UV light. The absorption of LED-emitted photons with wavelengths from 365 to 395 nm causes material electrons to transition to a high-energy state. Subsequently, it transitions to a low-energy state, along with fluorescence-emitting photons with wavelengths ranging from 430 to 450 nm. Given that the human eye typically responds to photons with wavelengths between 390 and 750 nm, we could detect the marker line. The wavelength of emission photons was slightly longer than that of absorption photons.

## Discussion

There are three common methods of marking the skin, namely, marker pens, henna, and permanent tattoos [6]. The marker ink pen affords a noninvasive and temporary marking method, although it has demerits such as fading and color migration. Temporary tattooing with henna, prepared from the mignonette tree, stains the superficial skin layers and yields stable markings for the period of external beam radiotherapy [7]. Permanent tattoos are widely used for skin marking in Europe and provide a permanent solid marking on the skin [8]. Recently, Goto et al. reported the first clinical use of a temporary fashion tattoo seal for skin marking during radiotherapy [9].

Several studies have compared fluorescent ink tattoos with conventional dark ink. Lim et al. conducted a prospective randomized trial to compare the reproducibility and body imaging of UV ink tattoos with those of dark ink tattoos in patients with breast cancer [10]. The authors reported no significant differences in the setup accuracy and body imaging between UV ink tattoos and dark ink tattoos. In addition, Landeg et al. reported a randomized controlled trial comparing fluorescent ink tattoos with dark ink tattoos in patients with breast cancer [11]. Six months post-radiotherapy, improved body imaging was achieved in the UV tattoo group when compared with that in the dark ink group, without reproducibility disorientation during radiotherapy.

Recently, SGRT has been used in skin marker-free patient setups. Giantsoudi et al. evaluated the accuracy of patient setup using a combination of SGRT and IGRT without reference to the tattoo arm compared with the tattoo arm at regional nodal irradiation of breast cancer [12]. They succeeded in accuracy in setup verification and reduction of treatment time without reference to tattoos. Naidoo et al. performed a systematic review of 13 full papers to evaluate the accuracy and reproducibility of the patient setup of the SGRT method compared to the conventional methods, such as permanent tattoo [13]. They concluded that SGRT could contribute to improving the accuracy and reproducibility of patient setup compared to the conventional method, which might reduce the frequency of IGRT to confirm the patient position. However, these treatment modalities are too expensive, and maintaining these also requires more budget. Our fluorescent pen marker is a simple marker-less implementation that is much less expensive and requires no equipment changes. The exact cost of our UV light system has not been undetermined, it would be at least significantly cheaper than purchasing a complete set of SGRT equipment.

This ink had been used as an evaluation of cleaning practices at a teaching hospital or permanent tattoo for the radiation therapy department. It seems to be safe, but long-term follow-up should be considered [5,11]. The wavelength of the UV light used in this study was classified as UVA (long-wavelength), which is generally considered far less carcinogenic than UVB or UVC. Moreover, UVA accounts for up to 95 % of the UV radiation reaching the earth [14] and is ubiquitous. Moreover, UVA phototherapy for several dermatological diseases was approved by the United States Food and Drug Administration [15]. Although UVA irradiation seems to be safe, careful long-term follow-up should be done.

## Conclusions

Even as IGRT has become the standard practice, skin markings are still used in many facilities as an indicator of patient positioning. Some areas of skin marking are difficult to conceal with clothing. Reducing or eliminating markings has a significant impact on patient satisfaction. Our approach might partially relieve the skin marking-induced discomfort during radiotherapy.

We propose the possibility of adapting UV fluorescent ink markers as invisible skin markers for radiation therapy. However, fading due to prolonged UV exposure remains a considerable limitation of fluorescent dyes. Further investigations are needed to estimate the utilization of fluorescent ink markers for radiation therapy in terms of safety, patient body imaging, reproducibility, and dye duration. Especially, the duration of the mark ink pen is one of the most important issues, which would be assessed under several conditions in future studies.
